# Spinning ice floes reveal intensification of mesoscale eddies in the western Arctic Ocean

**DOI:** 10.1038/s41598-022-10712-z

**Published:** 2022-04-29

**Authors:** Georgy E. Manucharyan, Rosalinda Lopez-Acosta, Monica M. Wilhelmus

**Affiliations:** 1 School of Oceanography, University of Washington, Seattle, Washington 98195 USA; 2 Department of Mechanical Engineering, University of California, Riverside, Riverside, California 92521 USA; 3 Center for Fluid Mechanics, School of Engineering, Brown University, Providence, RI 02912 USA; 4 Jet Propulsion Laboratory/California Institute of Technology, Pasadena, California 91109 USA

**Keywords:** Physical oceanography, Cryospheric science

## Abstract

Under-ice eddies are prevalent in the major circulation system in the western Arctic Ocean, the Beaufort Gyre. Theoretical studies hypothesize that the eddy-driven overturning and the ice-ocean drag are crucial mechanisms of the gyre equilibration in response to atmospheric winds. However, due to severe weather conditions and limitations of remote sensing instruments, there are only sparse eddy observations in the ice-covered Arctic Ocean. Hence, the evolution of the under-ice eddy field, its impact on the gyre variability, and their mutual response to the ongoing Arctic warming remain uncertain. Here, we infer the characteristics of the under-ice eddy field by establishing its tight connection to the angular velocities of isolated spinning sea ice floes in marginal ice zones. Using over two decades of satellite observations of marginal ice zones in the western Arctic Ocean, we identified and tracked thousands of floes and used idealized eddy modeling to infer the interannual evolution of the eddy energetics underneath the ice. We find that the eddy field is strongly correlated to the strength of the Beaufort Gyre on interannual timescales, which provides the major observational evidence consistent with the hypothesis of the gyre equilibration by eddies. The inferred trends over the past two decades signify that the gyre and its eddy field have been intensifying as the sea ice cover has been declining. Our results imply that with continuing sea ice decline, the eddy field and the Beaufort Gyre will keep intensifying and leading to enhanced transport of freshwater and biogeochemical tracers.

## Introduction

**Figure 1:**
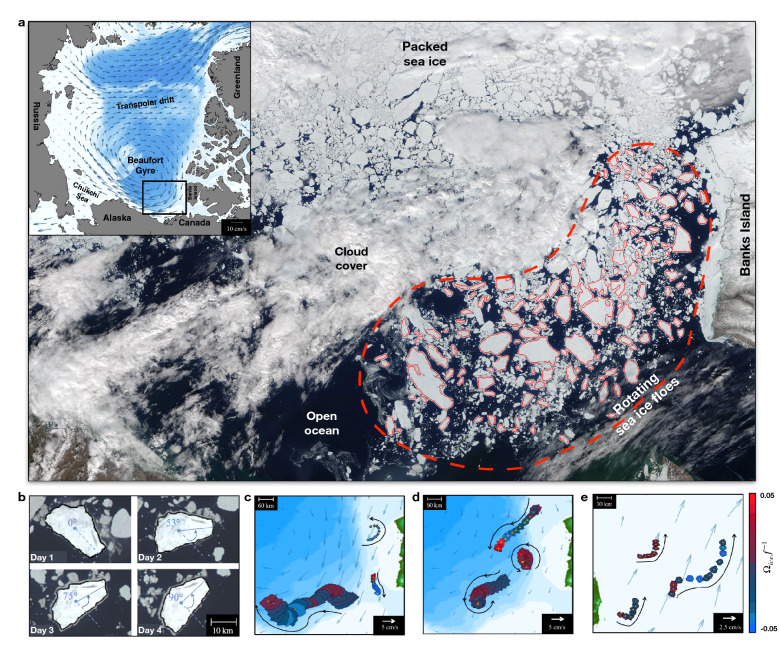
Sea ice floes and their rotation as seen in the marginal ice zone after the BG break-up event. (**a**) Representative MODIS True Color image (25.06.2008, downloaded from the NASA Worldview application) displayed in a WGS 84/NSIDC Sea Ice Polar Stereographic North 70” N projection. The red dashed line delineates the region of interest, whereby the contour of identified ice floes is outlined in red. The inset shows the Arctic Basin encompassing the BG and Transpolar drift and the area corresponding to the MODIS image (outlined in black). Superimposed with arrows is the sea ice velocity averaged over 1978–2020^49^. Ocean bathymetry is reproduced in color from ETOPO1, National Geophysical Data Center (NGDC) NOAA. (**b**) Time series of an ice floe displaying high angular velocities for four consecutive days (MODIS images from NASA Worldview application). (**c–e**) Examples of sea ice floe trajectories and angular velocities identified using the floe tracker algorithm^15^ (Methods). At a given acquisition timestamp, the color of each ice floe represents its angular velocity normalized by the Coriolis parameter.

The mechanisms governing the variability of the basin-scale anticyclonic circulation in the western Arctic Ocean—the Beaufort Gyre (BG) (Fig. 1a)—have been a matter of recent scientific debate. On the one hand, the eddy-driven overturning is thought to counteract the wind-driven strengthening of the gyre^1,2^. On the other hand, the sea ice cover can act as the dominant gyre equilibration mechanism by slowing down the currents via ice-ocean drag, leaving a minor role for eddies^3–5^. While the gyre state is likely somewhere in between these two limiting cases, it is expected that as the sea ice continues to retreat in the Arctic Ocean, the gyre will shift towards the eddy-governed regime^5^. Indeed, bulk estimates of the BG energy budget suggest that since 2008 eddies have been considerably draining potential energy and eventually dissipating it as kinetic energy at the ice-ocean interface^6^. However, the presence of sea ice in the Arctic Ocean imposes significant limitations on *in situ* field campaigns and hinders the acquisition of satellite eddy measurements common in temperate oceans. Without comprehensive eddy observations, projections of the BG transition to a climate with a dramatically reduced sea ice cover remain uncertain.

Here, we infer the BG eddy characteristics by taking advantage of the unique seasonality of its sea ice field. While the BG ice pack remains consolidated for most of the year, it naturally breaks up in late spring and summer^7,8^ due to repeating wind bursts and surface waves. As a result, vast BG areas are covered by ice floes behaving like brittle solid bodies floating over a turbulent ocean. The floes range dramatically in size, forming a power-law distribution that evolves seasonally and regionally between marginal ice zones and the pack ice^9–11^. Because of the overall thermodynamic melting that opens up ice-free ocean areas and the frequent atmospheric storms that pull the floes apart from each other, many floes at the edge of the BG marginal ice zone are weakly- or non-interacting. The BG region is unique since its broken-up floes are vast, $\mathcal {O}$(1–100) km in size, thus conveniently discernible from space, and, critically, comparable in scale to the underlying ocean eddies.

Central to our study is how trajectories of non-interacting floes reflect the atmospheric and oceanic stresses that drive their dynamics. The wind-driven motion of ice floes in free drift manifests in their unidirectional translation at a slight angle relative to the wind stress^12–14^. This component is dominant given the generally strong winds and low interactions between the floes in the BG marginal ice zone. Nonetheless, here we emphasize that the translation of isolated floes is accompanied by a considerable spin (*i.e.*, rotation around the center of mass as observed in Fig. 1), which has not been previously explored. We demonstrate below that floe rotation predominantly manifests the vorticity of underlying ocean eddies. We leverage this relationship to reconstruct the characteristics of the BG eddy field, infer its interannual variability, and establish its connection to the strength of the gyre and its changing sea ice cover.

## Results

## Observed sea ice floes and their rotation

**Figure 2:**
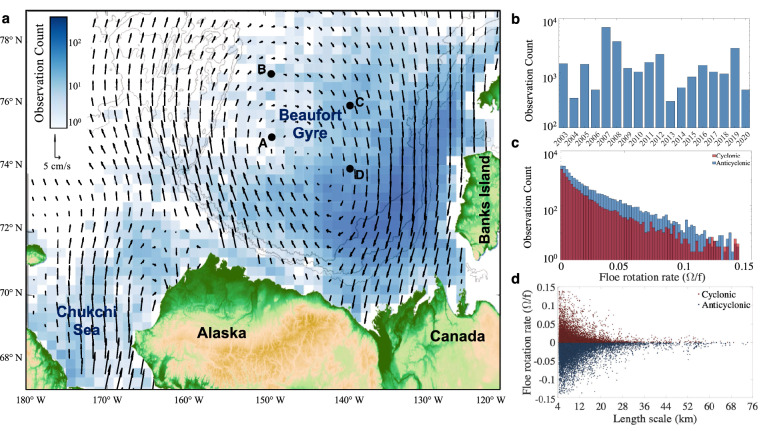
Statistical characteristics of the observed floe rotation rates. (**a**) Geographic distribution of the number of total ice floe observations plotted on a 25-km grid; the blue arrows denote the oceanic surface geostrophic circulation derived from sea surface height observations^42^; the gray contour lines denote the 500 m, 1500 m, and 2500 m isobaths. The black dots A-D indicate the locations of the moorings from the Beaufort Gyre Exploration Project (BGEP) used to validate our measurements of floe rotation rate variance. The figure was plotted using MATLAB version R2021a www.mathworks.com. (**b**) Net detected ice floe rotations per year. (**c**) Histogram of normalized floe rotation rates; cyclonic and anticyclonic rotation rates are shown separately in red and blue colors, respectively. (**d**) Normalized floe rotation rates against floe length-scale (square-root of the surface area).

By analyzing the sea ice field in the western Arctic Ocean for the past two decades, we produced a unique dataset containing thousands of floe observations and rotation rate measurements^15,16^. We used sequential optical satellite images (MODIS data at 250 m resolution) of the BG marginal ice zone to track individual floes and retrieve their spinning rates (Fig. 1). We retained only those that did not significantly interact with others and preserved their geometric shapes over at least two days (Methods). The frequent appearance of clouds in the images limits the number of sequential observations even in spring and summer when the BG sea ice breakup occurs. Nonetheless, we collected over twenty thousand rotation rate measurements for floes ranging from 4 to 75 km, spanning a large area of the Beaufort Gyre over the past two decades (Fig. 2). Most floes were identified in June and July in BG marginal ice zones after the consolidated winter sea ice shatters into smaller floes subject to strong wind and wave activity. In the following analysis, we treat these nearly non-interacting and shape-preserving floes as solid bodies, with their translational and rotational motion driven only by the oceanic and atmospheric stresses and torques.

**Figure 3:**
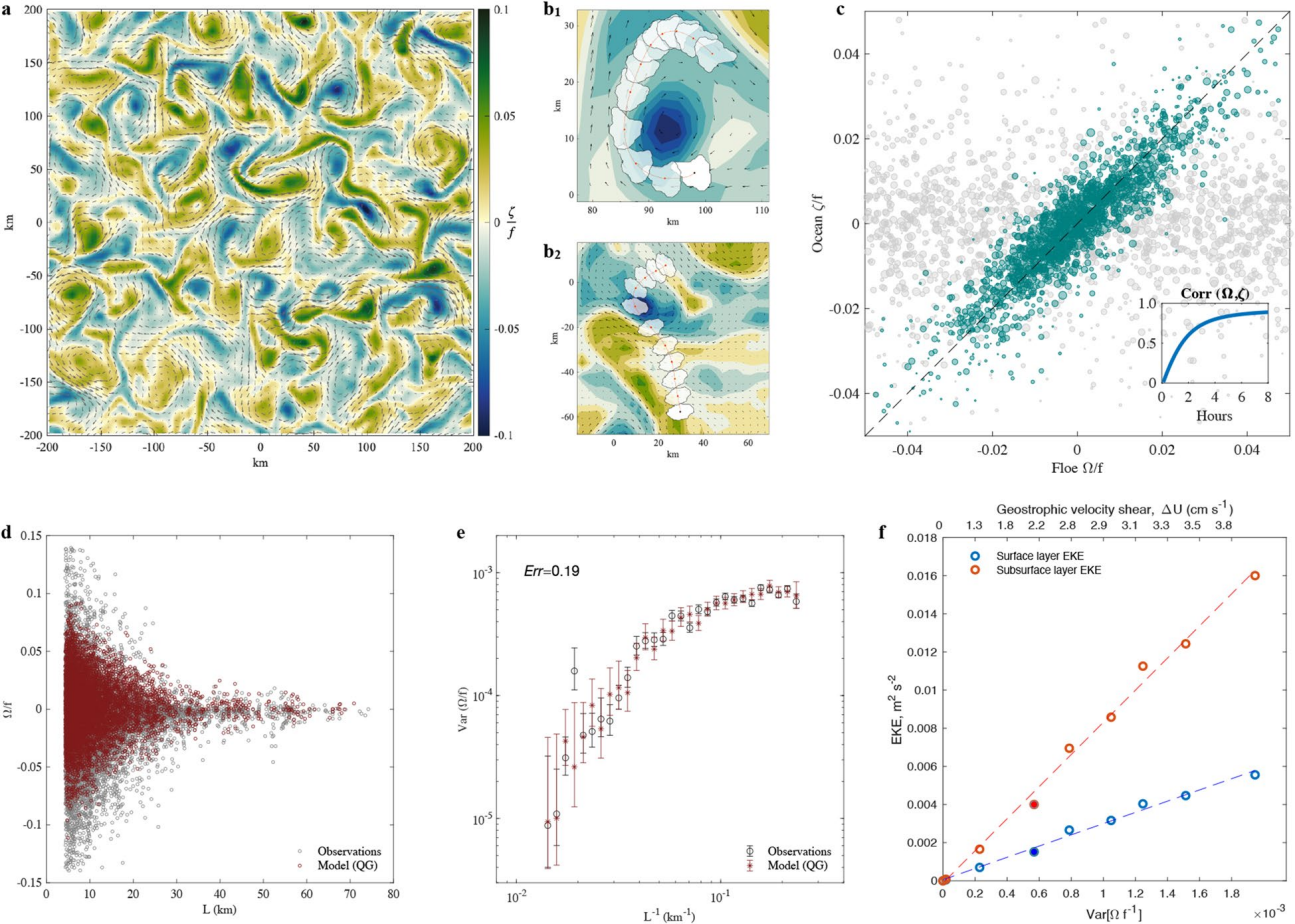
Simulated ice floe rotation over the best-fit eddy field. (**a**) A snapshot of the statistically equilibrated turbulent ocean eddy field generated by the QG model with best-fit parameters. (**b**) Examples of floe translation and rotation over eddies for the case of weak (**b1**) and strong (**b2**) winds. Panels (**a,b**) were plotted using MATLAB version R2021a www.mathworks.com. (**c**) Floe rotation rates plotted against the under-floe average ocean vorticity (both normalized by the Coriolis parameter *f*). Grey points represent the initial conditions with the random distribution of floe rotation rates. The green points represent the scatter once the dependence on the initial conditions is lost (after 8 h). The inset demonstrates the correlation between the floe-averaged ocean vorticity and the floe rotation rates plotted as a function of time. (**d**) Dependence of the normalized floe rotation rates on floe size, with gray points showing observations and red points showing the statistically equilibrated simulation. (**e**) Variance of the simulated (red stars) and observed (black circles) floe rotation rates as a function of floe length-scale; the error bars denote the 95% confidence intervals for estimating the variance assuming that rotation rates obey the normal distribution at each size bin. Note the data point with anomalously-high variance; most of the floes there are from 2007–2008, years with strong atmospheric forcing in the BG region. (**f**) The spatial and temporal EKE averages in the surface and subsurface layers of the QG model plotted against the normalized standard deviation of the simulated floe rotation rate variances driven by the model eddy field. The variances are computed based on all floes and hence dominated by the more frequent smaller floes. The filled circles represent the best-fit simulation given the observations in all available years. The top x-axis shows the values of the corresponding geostrophic velocity shear $\Delta U$ that was used to generate the synthetic eddy fields (note that $Var[\Omega ]$ increases monotonically but not linearly with $\Delta U$).

Typically, floes translate along one direction for multiple days (Fig. 1 c-e), due to the strong influence of large-scale atmospheric winds. Occasionally, when winds are low, the floes can be found rotating in nearly-circular motion (Fig. 1 c-e). More importantly, even in the presence of winds, the floes significantly spin around their centers of mass, with daily rotation angles changing in extreme cases by over 50°(Fig. 1b). During their nearly linear translation, the sense of rotation of the floes (cyclonic/anticyclonic) can change on characteristic length scales of about 10 to 40 km, and those switches can occur up to several times throughout a weekly period. The characteristic rotation rate of the observed floes, $\Omega$, depends strongly on their size (Fig 2). Normalized by the Coriolis parameter *f*, the rotation rates can reach values of up to 0.15 for the relatively small 5 km floes. In comparison, floes larger than 50 km rotate an order of magnitude slower than the smallest detectable floes (Fig 2d). Despite cyclones typically dominating the higher rotation rates in ice-free oceans^17^, the anticyclonically-rotating floes outnumber the cyclonic ones by about a 2:1 ratio (Fig 2c). This observed asymmetry in floe rotation is consistent with the predominance of anticyclonic eddies in the BG, one of the distinct features of its subsurface eddy field revealed by *in situ* field measurements^18–21^.

**Figure 4:**
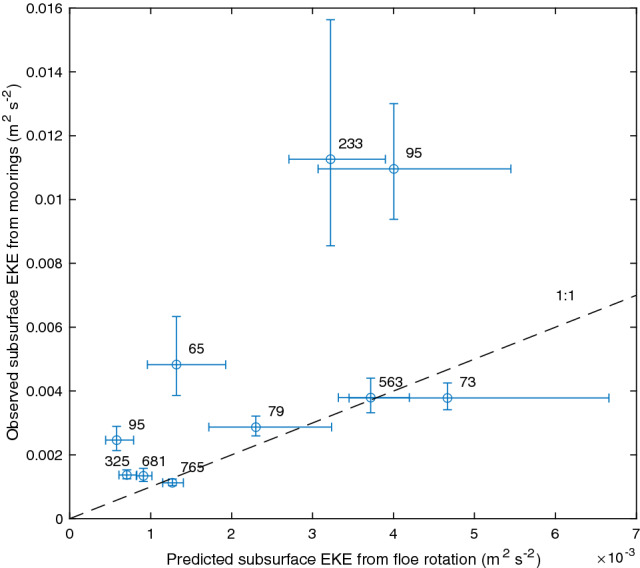
Comparison between the observed subsurface EKE from BG moorings A, C, and D (see Supp. Fig. 5,6) and the EKE inferred from floe rotation in the vicinity of those moorings (see Methods). The numbers denote the number of floes that appeared at a given year within a 200 km wide square box centered around the corresponding mooring location; EKE estimates are shown only for those years in which there were more than 50 floes in the box. The error bars represent the 95% confidence intervals in estimating the EKE from variances assuming a normal distribution for eddy velocities and floe rotation rates. The black dashed line represents the 1:1 relation. Note, the two outliers with extremely large observed EKE from moorings are associated with a combination of missing data during a significant part of the year coincident with overly strong eddies in mooring D in 2015–2016 (see discussion in Methods and Supplementary Figure 7). Excluding these two outliers, the observed and predicted subsurface EKE are significantly correlated with a coefficient of 0.55.

**Figure 5:**
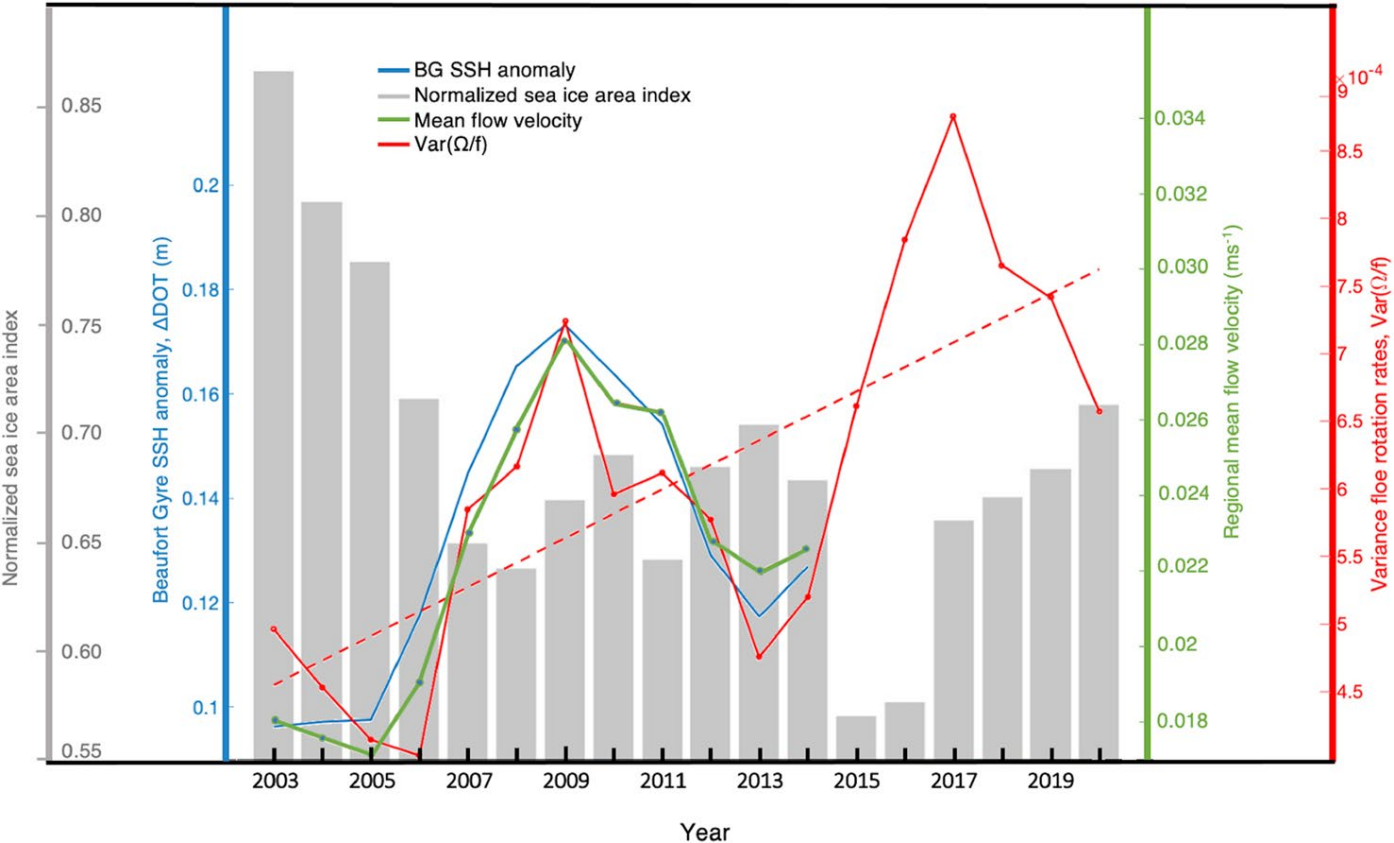
Interannual evolution of the bulk SSH anomaly associated with the BG surface geostrophic flow (blue), the mean geostrophic flow in the south-east region of the BG, where the majority of the observations were acquired (green), the variance of floe rotation rates as a proxy for EKE (red), and the seasonal mean of the sea ice concentration in the region (gray). The data points in the time series represent three-year running mean values. The linear trend over the entire observational record is plotted for the variance of floe rotation rates (dashed red line). See definition of ocean and ice indices in Methods. The sea surface height and corresponding geostrophic velocity data^42^ extends only until 2014.

Both atmospheric and oceanic stresses can, in theory, affect the angular velocity of ice floes. However, the observed magnitudes of angular velocities (Fig. 2b), persistence timescales (order of days), and frequent changes in the sense of rotation without changes in the direction of translation (Fig. 1c–e) strongly suggest that oceanic rather than atmospheric eddies dominate the observed rotation of floes in our study region. Strong atmospheric vortices are either more extensive than floes or pass them too quickly to deposit substantial torques and cause persistent floe rotation on a timescale of a few days. Some floe rotation may be induced by their complex three-dimensional shape, containing directional ridges^22,23^ that can effectively act as sails when the ice is drifting over the ocean. However, the sail effect cannot explain the frequent changes in the sign of floe rotation that are commonly observed without changes in the predominant direction of floe translation (Fig. 1c–e). Thus, the remaining rational explanation of the observed ice floe rotation is the presence of underlying ocean eddies, which happen to have similar characteristic length scales (5–100 km) and rotation rates (vorticity) as the floes^20,21,24,25^. As winds advect the ice floes over an eddying ocean, the eddy-induced ice-ocean torques can drive their rotation. In this case, the sense of floe rotation during its translational motion reflects the underlying cyclonic or anticyclonic eddies. Note that only eddies with a surface velocity expression affect sea ice motion.

## Inferred characteristics of the oceanic eddy field

We simulate the motion of ice floes as solid bodies forced by atmospheric winds and oceanic stresses. To this end, we use a reanalysis product^26^ and synthetic eddy fields generated by a quasigeostrophic (QG) model (see Methods and Supplementary Material). The simulated translational and spinning motion of floes is qualitatively consistent with the observations (Fig. 3). At relatively low winds and in the presence of strong eddies, the simulated floe trajectories curve around underlying eddies and the floe angular velocities are consistent with the sign and magnitude of the eddy rotations (a representative case is shown in Fig. 3b1; also see Supplementary Video). When winds are strong, the motion of the floe is predominantly in one direction. However, as it passes over various eddies, it spins consistently with the eddy rotation (Fig. 3b2). Importantly, it only takes a few hours for the ice floes to completely lose their dependence on initial conditions and reach statistical equilibrium with the driving forces (Fig. 3c). As a consequence of the strong ice-ocean coupling, the floe angular velocity is highly correlated to the ocean vorticity averaged over the floe surface area (Fig. 3c, inset). Considering the daily timescales of sequential floe observations, the model equilibrates rapidly, implying that the ice floe rotation rates are in quasi-equilibrium with the underlying oceanic eddy field.

By tuning the two-layer QG model parameters, we found the eddy field characteristics that lead to the best fit between the observed and simulated rotational statistics of floes, specifically matching the rotation rate variances across all floe length scales with an average error of 19% (see Methods and Fig. 3d–e). The inferred best-fit model parameters provide insight into the eddy characteristics, suggesting the eddies are located only in the upper halocline. The best-fit Rossby deformation radius ($R_d = 5.6$ km) closely matches the observed second baroclinic mode radius, not the first, which is about two times larger in the BG^27^. The pertinence of the second baroclinic mode radius is consistent with the vertical profile of observed BG eddies that are localized within the halocline and do not extend all the way to the bottom of the ocean^19–21^. Note, eddies of purely the first baroclinic mode structure would have a velocity maximum near the surface and extend to the bottom, with only a single zero-crossing of the velocity occurring within the halocline. Most of the observed BG eddies are localized in depth and are composed of several dominant baroclinic modes^28^, which rationalizes why our two-layer QG model has a strong preference for the second baroclinic mode radius instead of the first one. The inferred ratio of the layer depths is close to one, which is significantly higher than the $\mathcal {O}$(0.06) value corresponding to eddies interacting with the abyssal layers. The best-fit velocity shear of about 2 cm/s is consistent with estimates from BG moorings and the observed surface geostrophic flow of 2–3 cm/s in the BG interior^29^. Thus, the consistency between the estimated best-fit parameters and the observed stratification and mean flow shear supports the notion that eddies were formed due to local baroclinic instabilities. Nonetheless, it is also important to note that our floe-based method reconstructs effective characteristics of all ocean eddies that have surface expressions. Hence, some of the under-ice eddies may have formed remotely (e.g., near continental slopes) and drifted to the BG interior as has been previously hypothesized^19,30^.

Our novel method resulted in eddy field characteristics that are in many ways consistent with observations from various measurement platforms as well as with large-scale BG energy constraints. The surface eddies are abundant, ranging from 10 to 60 km in size with characteristic Rossby numbers of $\mathcal {O}$(0.1) (Fig. 3a). While the $\mathcal {O}$(10) km eddy scales are consistent with sparse *in situ* observations^21^, our results point to the existence of relatively large eddies (50 km and larger). Such large BG eddies have been recently identified in remote sensing imagery from their signatures on the sea ice distribution in marginal ice zones^24^ as well as from open-ocean altimetry^25^.

The inclusion of the quadratic ice-ocean drag played a crucial role in finding the optimal eddy field. Consequently, in contrast with ice-free oceans, the dominant mechanism of eddy energy dissipation in ice-covered oceans is at the surface, not at the bottom. Our best-fit model allows to estimate that the dissipation of eddy kinetic energy (EKE) by the sea ice-ocean drag occurs at a rate of about 0.7 mW m$^{-2}$. To put this number into perspective, consider the magnitudes of sources/sinks in the large-scale energy budget of the BG^6^. During the 2008–2014 period, the rate of kinetic energy input to geostrophic BG currents was about 0.1 mW m$^{-2}$ but can reach about 1 mW m$^{-2}$ in exceptional years like 2007^6^. The missing energy dissipation rate needed to close the BG energy budget in the 2007–2014 period was about 0.1-0.2 mW m$^{-2}$. The source was hypothesized to be mesoscale eddies that form in the interior of the BG halocline and drain the available potential energy (APE) stored in large-scale BG currents^6^. Another way of gauging the importance of eddies is to compare the gyre equilibration timescale with the time eddies would take to drain the excess APE gained during the gyre spinup. The APE variation over the 2003–2014 period was estimated at 40 PJ^6^. At a rate of 0.7 mW m$^{-2}$ uniformly spread over the BG area of about 10$^6$ km$^{2}$, the eddies are expected to drain the excess APE entirely in only two years. This eddy-driven APE drainage timescale is significantly shorter than the nearly decade-long gyre equilibration timescale^31,32^, highlighting the major role of eddies in BG energy cycle and equilibration.

The eddy field thus has a subsurface kinetic energy maximum, with the top layer EKE of 0.0017 m$^2$ s$^{-2}$ and the bottom layer EKE of 0.0045 m$^2$ s$^{-2}$ (Fig. 3f). The EKE levels are in quantitative agreement with *in situ* observations from moorings A,C, and D that are away from bathymetric features (Supplementary Fig. 5b). The surface and subsurface EKE in the QG model are both linearly proportional to the variance of the simulated floe rotation rates (Fig. 3f), and this relation can be used to estimate EKEs from floe observations. We perform a validation test by comparing the observed EKE at mooring locations and the EKE predicted by the floe rotation rate variance. Since we are confined to a small domain centered at the location of each mooring (a square box of 200 km in size), there are only a few years where both a sufficient number of floe and mooring observations are present (Supplementary Fig. 6). It is important to highlight that the mooring EKE observations were not assimilated in the QG model and are thus an independent dataset. Apart from a few outliers (discussed in Methods), the predicted and observed EKE levels agree quantitatively with a correlation coefficient of 0.55 (Fig. 4). The agreement between the crucial characteristics of the inferred eddy field and existing sparse observations from different measurement platforms supports our hypothesis that the statistics of floe rotation predominantly reflect the properties of the oceanic eddy field.

## Interannual BG variability and decadal trends due to sea ice decline

The reconstruction of floe rotation rates over the 2003–2020 period allows us to identify the relation between the strength of eddies and large-scale BG currents and assess their interannual and decadal trends (Fig. 5). In comparing the time-evolution of eddy and mean flow indices, we used a three-year running-mean filter to reduce noise and account for the eddy memory effect^33^, the time needed for an eddy field to respond to the time-dependent mean flow. We find that over 2003–2014, when the satellite-derived observations of geostrophic ocean currents were available, the interannual variability of ice floe rotation rates (a proxy for EKE) matches well with the BG strength index and the magnitude of the regional currents; correlations between pairs of indices range from 0.83 to 0.9. The low energy years for the eddy field were 2005–2006 and 2013, while 2008–2010 and 2016–2018 were the high energy years. The tightly correlated interannual variability of the eddy field and the BG strength supports the eddy equilibration hypothesis. However, recent moderate-resolution numerical simulations of the wind-driven BG spinup suggest that its eddy field does not significantly change in response to the spinup of the gyre^34^, which contradicts our observations. This incongruence implies that even comprehensive general circulation models do not adequately simulate eddy formation processes in the BG, significantly underestimating EKE likely due to inaccurate representation of the stratification and vertical shear of the large-scale currents.

The presence of sea ice and its associated ice-ocean drag is crucial in constraining the evolution of the large-scale BG currents and its eddy field. On interannual timescales, the gyre indices and the floe rotation follow opposite trends with the sea ice concentration (Fig. 5). Broad trends in over two decades of observations revealed a twofold increase of the floe rotation variance as the average sea ice concentration decreased by roughly 30%, implying that the eddy field has been intensifying as well with a similar twofold EKE increase.

## Discussion

This study presents a unique method of inferring the under-ice eddy variability in the western Arctic Ocean by analyzing the motions of relatively isolated sea ice floes in marginal ice zones. Specifically, using theoretical arguments and idealized modeling, we deduced that the scale-dependency of the floe rotation rate is directly related to the characteristics of the underlying oceanic eddy field. Piecing together almost two decades of satellite floe observations with a physical model of eddy dynamics, we inferred the best-fit characteristics of the ocean eddy field, including eddy lengthscales, EKE, and dissipation rates.

Our method was validated with independent *in situ* observations of subsurface EKE at the four BG mooring locations. Since our estimated subsurface EKE levels agreed well with mooring observations, it is reasonable to conclude that the surface eddies driving floe rotation extend into the halocline layer, *i.e.*, these are not predominantly mixed layer eddies. Note, marginal ice zones are known to have strong density gradients that support surface eddy formation via frontal instabilities^35,36^. However, most of our floe observations are taken shortly after the consolidated pack ice breaks up into smaller floes, and hence surface density gradients have not been fully developed. Since the eddy field requires some time to adjust to the relatively ice-free conditions after the sea ice breaks up in spring/summer, our estimated eddy characteristics are likely more attributable to winter-like eddy fields formed under consolidated sea ice. Highly concentrated winter sea ice can significantly suppress mixed layer instabilities and surface eddies^37,38^. Thus, the expected lack of mixed layer eddies implies that floes are rotated predominantly by eddies that have origins in the interior of the ocean, not at the surface. This rationalizes the good fit between the observed subsurface EKE and that estimated from floe rotation that depends on surface eddies.

Our analysis unveiled the ongoing under-ice eddy response to climate change in the western Arctic Ocean and demonstrated its tight connection to the BG currents and sea ice cover. The critical finding of our study is that the eddy field has been responding rapidly to changes in sea ice, with decadal trends indicating that the floes and underlying ocean eddies are now spinning significantly faster than two decades ago. The presence of sea ice dramatically suppresses the eddy field as its EKE is dissipated by friction due to the ice-ocean drag. As a result, the large-scale currents and the eddy field in the region can be expected to become much more energetic given the observed trend in sea ice extent. To this day, it had not been possible to reach this conclusion using existing *in situ* eddy observations because their spatial or temporal coverage is very sparse given the heterogeneity and interannual variability of the eddy field. We note that existing mesoscale and submesoscale eddy parameterizations were developed and calibrated based on ice-free theory and modeling^39,40^. They do not account for additional EKE dissipation at the ice-ocean interface and hence likely overestimate eddy diffusivities and associated overturning in ice-covered regions. As global warming drives the Arctic Ocean towards a seasonally ice-covered state, developing improved eddy parameterizations is particularly important to accurately capture changes in eddy dynamics, their impact on large-scale currents, and distribution of water masses in ice-covered regions.

The hypothesis regarding the intensification of the BG eddy field with declining sea ice cover has been put forward based on the analysis of sources/sinks of gyre mechanical energy from 2003 to 2014^6^. It was estimated that from 2008 to 2014, the gyre was storing less potential energy relative to the kinetic energy it was receiving from combined wind and sea ice stress work. The apparent energy surplus can be explained by the intensifying energy transfer from the BG potential energy via interior baroclinic instabilities towards eddies and the eventual dissipation of EKE by sea ice-ocean drag. The conclusion that the increasing imbalance between the cumulative surface-stress work and the available potential energy is a signature of increasing eddy energy relied crucially on the hypothesis that the gyre is significantly equilibrated by eddies. However, the lack of comprehensive eddy observations questions the conclusion about the possible eddy intensification. Our study of floe rotation provided the much-needed independent estimate of the BG eddy field characteristics under sea ice, right where the dissipation of eddy kinetic energy occurs. Our comprehensive analysis spanning observations from 2003 to 2020 concludes that the BG eddy intensification is indeed ongoing. It is not simply a part of interannual gyre variability but rather represents a strong decadal trend in response to the continuing sea ice loss in the western Arctic Ocean.

Finally, we comment on the implications of our results for the ongoing debate on the mechanisms of gyre equilibration. We provided the first observational evidence linking the EKE to large-scale BG currents and established that the EKE dissipation at the ice-ocean boundary layer is an important term in the BG energy budget. These results are consistent with the eddy equilibration hypothesis. They imply that the sea ice-ocean drag on its own does not entirely equilibrate the gyre, even though it does significantly reduce the Ekman pumping and the kinetic energy input into the gyre. These conclusions come with a noteworthy caveat that our method does not distinguish the eddy formation mechanisms and reconstructs combined characteristics of all eddies with surface expressions capable of driving floe rotation. To better understand the role of mesoscale eddies in the BG equilibration and interannual variability, it is necessary to quantify the eddy formation mechanisms and the associated eddy overturning, focusing on eddies formed via baroclinic instabilities of the large-scale currents in the BG interior.

## Methods

### Datasets

- Satellite Imagery. Sea ice measurements are retrieved from Moderate Resolution Imaging Spectroradiometer (MODIS) optical imagery (Level 1B 250M). This dataset can be directly downloaded from the open-access Earth Observing System Data and Information System (EOSDIS) Worldview platform (https://worldview.earthdata.nasa.gov). In this study, both Corrected Reflectance True and False Color images spanning spring and summer from 2003 to 2020 are employed. The reader is referred to the work of Lopez-Acosta et al.^15^ and Wolfe et al.^41^ for specifications regarding satellite acquisitions.
- Ocean SSH. We employ measurements of monthly dynamic ocean topography (DOT) and geostrophic velocity currents in the Arctic basin retrieved by Armitage et al.^29,42^. These measurements combine SSH estimates from open and ice-covered waters derived from Envisat (2003–2011) and CryoSat-2 (2012–2014).
- Atmosphere. The effect of atmospheric forcing is assessed using ERA5 10 m wind speed reanalysis data (available with a temporal resolution of 1 h and a spatial resolution of 0.25$^{\circ }$). The product is distributed by the European Centre for Medium-Range Weather^26^.
- Sea Ice Area Index. It is defined as the area covered by sea ice relative to the total area of the Beaufort Sea (983,663 km$^2$). It is obtained from the National Sea Ice Data Center (NSIDC) Sea Ice Area Index data product^43^ at 25 km with daily temporal resolution.
- Sea Ice Concentration. We evaluate sea ice concentration to discard interactions between identified ice floes. It is obtained from the NSIDC product MASAM2^44^. Daily concentrations are obtained at 4 km resolution by blending observations from the Multisensor Analyzed Sea Ice Extent (MASIE) and the Advanced Microwave Scanning Radiometer 2 (AMSR2) products.
- Beaufort Gyre Moorings. Mooring velocity measurements are analysed to estimate the strength of mean currents and the subsurface eddy kinetic energy. The data (and its description) from the four BG moorings named A, B, C, and D (locations shown in Supp. Fig. 5) can be downloaded from the Beaufort Gyre Exploration Project website: https://www2.whoi.edu/site/beaufortgyre/data/mooring-data/.

### Oceanic flow indices

- Regional mean flow velocity. We characterized the strength of oceanic and atmospheric forcing in the BG from kinetic energy (KE) estimates. The seasonal oceanic and the wind surface KE is calculated as $KE_o=\frac{1}{2}\rho _o \big (<u_o^2>+<v_o^2> \big )$ and $KE_w=\frac{1}{2}\rho _w \big (<u_w^2>+<v_w^2> \big )$, respectively. Here, the monthly geostrophic oceanic currents are denoted by ($u_o$, $v_o$), and daily 10 m wind currents are denoted by ($u_w$, $v_w$). Angle brackets denote regional averages within the time frame of availability of sea ice floe observations. Wind and water density values are taken as $\rho _w$=1 kg/m$^3$ and $\rho _o$=1000 kg/m$^3$. A seasonal metric for each type of forcing is produced by averaging the KE annually over each season.
- BG SSH anomaly. To characterize the strength of the BG, we calculate the characteristic sea surface height difference between an area encompassing the center of the gyre and its periphery. The center of the gyre is defined as the region enclosing most of the gyre during the spring and summer seasons from 2003 to 2020 (delineated by a red outline in the Supplementary Material). The area surrounding the gyre excludes shallow waters that are below 100 m deep (Fig. 1b).

### Ice floe identification and rotation rate measurements

We identified and tracked ice floes using a newly developed feature matching algorithm that analyzes Moderate Resolution Imaging Spectroradiometer (MODIS) imagery from the EOS-NASA satellites, Terra and Aqua^15^. In particular, two pairs of digital color images (Daily Corrected Reflectance True Color and Daily Corrected Reflectance False Color, with 250 m resolution) are analyzed per satellite to extract sea ice features amid changing atmospheric conditions. The process consists of three main modules: image processing, feature identification, and sea ice tracking.

The image processing module effectively minimizes noise due to uneven illumination and cloud coverage while increasing the contrast between ice floes and ocean water. Geometrical parameters are then extracted, including perimeter, major and minor axes of the ellipse that matches the second central moment of the floes, centroid position, and surface and convex areas. The convex area is defined as the area of the smallest convex polygon enclosing an ice floe. As a result, ice floes with length scales ranging from 4 to 75 km are readily identified in each image.

Ice floes are tracked by (1) comparing the suite of geometrical parameters of the ice in successive images, (2) finding potential matches, and (3) selecting the best candidates based on the assessment of a similarity metric and the surface area difference between them. The rotation rates of tracked ice floes are calculated by superimposing the shapes of ice floe pairs, rotating one over its axis, and tracking the angle change until the overlapping area is minimal. Finally, the angular velocity of each ice floe is calculated by averaging Aqua and Terra outputs and taking into account the timestamp at which the MODIS images being analyzed were acquired.

The last step in creating our library of floes and their rotation rates is identifying and retaining the relatively isolated floes, i.e., those that do not significantly interact with nearby floes. This ensures that ice floe rotation is primarily due to the effect of external atmospheric and oceanic stresses rather than collisions. To achieve this, we computed the bounding box of identified ice floes. Interacting floes were detected based on the proximity of their respective bounding box and the instantaneous value of their rotation rates. Finally, sea ice concentration was evaluated (from MASAM2) to discard any identifications in regions with concentrations higher than 90$\%$. Due to the relatively coarse resolution of satellite products, some retained floes may be surrounded by much smaller unidentified floes and hence are not perfectly isolated (Fig. 1b). Potential interactions with surrounding smaller floes would increase the effective floe size, introducing small biases towards underestimating the reconstructed eddy length scales and/or vorticity. Our algorithm for retaining only the non-interacting floes minimizes these biases but substantially reduces the number of observations. Yet, we tracked the motion of more than twenty thousand isolated floes in the Beaufort Gyre over an entire observational period (2003–2020), with most floes identified in June and July.

### Evolution of floes subject to oceanic and atmospheric stresses

The statistical characteristics of ice floe rotation rates are obtained by evolving the floes with observed shapes as solid bodies subject to forces and torques due to ice-ocean and atmosphere-ice stresses. No collisions between the floes are simulated as the statistics are compared against those of observed non-colliding floes. The ocean- and atmosphere-ice stresses are parameterized using quadratic drag laws as:

1 $$\begin{aligned} \varvec{\tau _o} = \rho C_{do} |{\mathbf {u}}_o-{\mathbf {u}}_i|(\mathbf{u}_o -{\mathbf {u}}_i) \;\;\text {and}\;\; \varvec{\tau _a} = \rho C_{da} |{\mathbf {u}}_a-{\mathbf {u}}_i|({\mathbf {u}}_a - {\mathbf {u}}_i) \approx \rho C_{da} |{\mathbf {u}}_a| {\mathbf {u}}_a, \end{aligned}$$

where ${\mathbf {u}},{\mathbf {u}}_a,{\mathbf {u}}_o$ are the local velocities of sea ice, atmospheric winds, and ocean currents, respectively, and typically $|{\mathbf {u}}_a| \gg |{\mathbf {u}}_i|$. Only the two-dimensional motion of floes in the horizontal plane is considered. The sea ice velocity includes components due to the angular velocity, $\Omega$, and translational velocity of the center of mass ${\mathbf {u}}_i$:

2 $$\begin{aligned} {\mathbf {u}}({\mathbf {r}}) = {\mathbf {u}}_i + \Omega \hat{ \mathbf{k}} \times ({\mathbf {r}}-{\mathbf {R}}_i), \end{aligned}$$

where $\hat{{\mathbf {k}}}$ is the unit vertical vector, ${\mathbf {r}}$ is the horizontal coordinate vector, and $\mathbf{R}_i$ is the horizontal vector denoting the center of mass of an ice floe. The angular and the center-of-mass velocities are evolved according to momentum conservation laws involving atmospheric and oceanic stresses as well as the Coriolis force and the tilt of the sea surface height associated with ocean currents:

3 $$\begin{aligned} M \left( \frac{d {\mathbf {u}}_i}{dt} + f {\mathbf {k}} \times u_i \right) = \iint _A \left( \varvec{\tau _a}+ \varvec{\tau _o} - M g \nabla \eta \right) dA,\\ I \frac{d{ \Omega }}{dt} = \iint _A \mathbf {(r-r_i)} \times \left( \varvec{\tau _a}+ \varvec{\tau _o} - M g \nabla \eta \right) dA, \end{aligned}$$

where $M=\rho _i A h$ is the floe mass, $\rho _i$ its density, *A* its surface area, *h* its characteristic thickness, *I* its moment of inertia, ${\mathbf {r}}$ is the radial vector with origin at the position of the floe center of mass, *g* is the acceleration due to gravity, $\eta$ is the sea surface height anomaly associated with ocean currents, and $\nabla =(\partial _x, \partial _y)$ is the horizontal gradient operator.

The ice floe area is estimated from the library of geometrical properties obtained from MODIS imagery^15^. An Eulerian grid with a resolution of 250 m is defined in the coordinate system moving with the center of mass of a rotating ice floe and used to calculate the surface integrals of forces and torques. Floe thickness observations are not available in the BG, but floes are expected to be between 0.5 to 2 m thick during the breakup events of the winter ice pack. Even though the equilibration timescale is sensitive to the thickness of the floes, it ends up being relatively short (ranging from one to a few hours), implying negligible inertial terms in Eq. 3. Ice floes thus evolve such that all torques and forces balance each other out on daily timescales. Consequently, the choice of ice floe thickness does not significantly affect estimates of the rotational statistics. As a result, ice floes are in quasi-equilibrium with the underlying eddy scales on the timescale of $\mathcal {O}$(days), relevant to the observations. Here, we choose a constant characteristic ice thickness for all floes of 1 m.

The oceanic mesoscale flow, ${\mathbf {u}}_o$, is simulated with the QG model described in the following section. Each floe has a mask and an associated Eulerian grid onto which the ocean velocity is interpolated to calculate the stresses and torques as surface integrals. We assume a homogeneous atmospheric velocity field because in a reference system following the ice floe and considering scales of $\mathcal {O}$(10 km) and 24 h, atmospheric winds are not expected to have a considerable rotational component as to induce a significant rotation of the flow with respect to the floe rotation induced by ocean eddies. Trajectories for each of the observed floe shapes are calculated independently by randomly initializing their locations with respect to the ocean eddy field and randomizing their initial drift and angular velocities with a standard deviation matching that of the observations. For each floe, uniform atmospheric winds have been applied, either a constant value for sensitivity experiments or the one taken from the atmospheric reanalysis for the specific date and location of the floe. The model output contains the floe center of mass trajectory and velocity as well as its angular velocity; the values are taken at *t*=1 d since the start of the simulations after the dependence on the initial floe conditions is completely lost. For a given oceanic flow corresponding to a snapshot of equilibrated mesoscale turbulence, floe rotation rates are recorded, from which the histogram of rotation rate variance is computed as a function of floe size for quantitative comparisons with observations.

### Quasigeostrophic model of mesoscale eddies

We use an idealized two-layer quasi-geostrophic (QG) model^45,46^ to identify an eddy field that reproduces the observed rotational statistics of ice floes. The QG model generates eddies from baroclinic instabilities of vertically-sheared horizontal currents, which is the most common eddy-formation mechanism in the ocean^47^. The simplicity of the QG model is matched by its flexibility to generate a wide range of eddy field characteristics by varying only a few of its parameters: the Rossby deformation radius, $R_d$, the vertical shear of the horizontal currents, $\Delta U$, the ratio of layer depths, $\delta$, and the drag coefficients with the top and bottom boundaries (Supplementary Material). The model uses an f-plane approximation because there is a weak beta effect at high latitudes of the BG. The equations are solved numerically using a spectral method^46^ within a square doubly-periodic domain of 400 km by side and 256 spectral modes (resolution of about 1.5 km). The spectral filtering is applied for the smallest (grid size) eddies in the domain to halt the forward enstrophy cascade and ensure numerical stability.

The simulated background flow becomes unstable and generates mesoscale eddies that actively interact with each other, developing a homogeneous and isotropic turbulent eddy field with a clear inertial range of the kinetic energy spectrum. The production of eddy kinetic energy is balanced by its frictional dissipation, predominantly by the ice-ocean drag. A commonly used quadratic ice-ocean drag law (with the coefficient $C_d = 0.0055$) is imposed at the top layer, providing a crucial energy dissipation mechanism for the eddies. Note, the linear ice-ocean drag formulation leads to an unrealistically large equilibrated kinetic energy and characteristic Rossby numbers well above one. Due to the prevailing inverse energy cascade in QG turbulence, eddy-eddy interactions favor the formation of large-scale eddies. However, the largest possible eddy scale is constrained by the quadratic drag that dissipates the eddy energy well before its scale reaches the domain size.

The QG model generates only geostrophically-balanced eddies and does not represent other relatively fast-evolving unbalanced motions like inertial oscillations, internal gravity waves, or surface frontal features. However, since even for the smallest observed floes (about 5 km in size), the rotation rate $\Omega /f\sim \mathcal {O}(0.1f)$, the ice floe rotation observed with daily time intervals would predominantly reflect the relatively slower geostrophically-balanced ocean currents. The unbalanced inertial oscillations and near-inertial internal gravity waves can be energetic in marginal ice zones. Nonetheless, their average impact would only amount to a small noisy contribution given their short oscillation period (about 12 hours at high latitudes) relative to the observed floe angular velocity persistence time scale (of a few days).

### Estimation of crucial QG model parameters

The key adjustable parameters of the QG two-layer model include the bulk vertical shear of the background horizontal velocity $\Delta U$, the deformation radius $R_d$, and the effective ratio between the top and bottom layer depths $\delta$. We explored the sensitivity to those parameters to identify the best fit of the simulated and observed scale-dependency of the floe rotation variance. The modeled rotation rates are those obtained by evolving the observed shapes of floes over a field of ocean eddies generated using a set of model parameters ($\Delta U, Rd, \delta$). The rotation rates are then compared to the observed rotation rates statistically, using a quantitative loss function, *Err*, defined as

4 $$\begin{aligned} Err= mean \left( \frac{|var(\Omega _{model})-var(\Omega _{obs})|}{var(\Omega _{obs})}\right) \;, \end{aligned}$$

where the rotation rate variances are computed for each size bin after which their absolute values are averaged over all bins. The loss function normalizes rotation rate variance error by the observed variance, and hence it optimizes the fit over all the floe sizes. The loss function was minimized starting from an initial guess of $\Delta U=3$ cm s$^{-1}$, $Rd=13$ km, $\delta =0.1$ by continuously optimizing a single parameter at a time in the order ($Rd, \delta , \Delta U$), until the best-fit parameter set $\Delta U=1.8$ ms$^{-1}$, $Rd=5.2$ km, and $\delta \approx 1$ minimized the loss function to $Err\approx 0.19$. This best-fit implies that the corresponding eddy field leads to simulated rotational variances of floes that, on average, match the observations to within 19% accuracy for all observed floe sizes, ranging from 5 to 80 km.

The three QG model parameters dramatically affect the corresponding eddy field and hence the rotational statistics of the simulated floes. The magnitude of the vertical velocity shear affects the strength of the eddies and hence variances of floe rotations at each length scale, moving the variance–length-scale curve (Fig. 3e) vertically on a log scale. For a given velocity shear, changing the deformation radius and the ratio of layer depths affects the overall energy of eddies and, more importantly, the scales of the equilibrated eddies and hence the slope of the variance-lengthscale curve. Specifically, increasing *Rd* or decreasing $\delta$ leads to an increment of the eddy scale and reduced energy and Rossby numbers. The choice of $R_d$ and $\delta$ values is inherently linked to the vertical structure of eddies and the type of instabilities being generated. Suppose the observed floe rotations reflect the surface expressions of first baroclinic mode eddies, resulting from an instability that involved the interactions between the upper halocline (about 200 m thickness) and the deep ocean (about 2–4 km thick layer). In that case, the appropriate values are $R_d=13$ km (first Rossby deformation radius) and the ratio of depths $\delta \sim \mathcal {O}(0.1)$. However, given a characteristic range of the velocity shears of $\Delta U \sim \mathcal {O}(0.02)$ m/s, these parameters lead to an unrealistically weak eddy field with $Ro \sim O(10^{-3})$ and length scales $\mathcal {O}$(300 km) that equilibrate on multi-decadal timescales. This implies that the baroclinic instability that leads to eddy formation is likely occurring in the upper ocean without involving the rest of the abyssal ocean. Thus, reducing the deformation radius and increasing the layer thickness ratio is necessary to adjust the characteristics of the eddy field. Since *Rd* and $\Delta U$ are only related to the background stratification (and perhaps the location of the relevant vertical velocity shear) but not to the strength of the mean currents, we keep the optimal values of these parameters constant. We assume that the observed interannual variability in the floe rotational variance is dominated by the changes in the magnitude of the velocity shear. Note, the observations only give the surface velocity *U*, while the effective velocity shear $\Delta U$ represents the difference between the bulk velocities in the upper layer and the layer below it. However, from mooring observations at a nearby location, it is clear that the velocity profile is significantly surface-amplified and hence *U* is only a slight overestimate of $\Delta U$.

### Estimation of the subsurface EKE based on the variance of floe rotation rates

To validate our results that the statistics of floe rotation contain direct information about the oceanic eddy field, we compare the predictions of subsurface EKE from the floe statistics with the point EKE measurements from BG moorings. The EKE from floe rotation is calculated as $\alpha Var(\Omega /f)$, where $\alpha =8.5$ m$^2$ s$^{-2}$. This constant was obtained from the linear fit between the subsurface EKE and the variance of floe rotation rates for simulated floe motion over synthetic eddy fields from the idealized QG model (Fig. 3f). The variance of floe rotation rates is calculated for floes that have centers of mass within a 200 km box centered at the location of a mooring. The observed EKE from the moorings is calculated by first taking an average of horizontal velocities over a depth range of 100 to 200 m, and then taking annual averages for each available observation year. In the subsurface layer, there is only a weak seasonal cycle of turbulence which rationalizes taking year averages for mooring EKE to construct a metric that is more representative of an average over an area in some vicinity of the mooring location. Note that the mooring EKE represents a point measurement while the EKE inferred from floes is more representative of an average over a 200 km box. While there is a large number of floe observations available over the entire BG region, there is a dramatically smaller number of floes in relatively small boxes (200 km wide) centered at any particular mooring location. Hence, it is not possible to get floe-derived EKE estimates for all moorings at all years (Supplementary Fig. 6). We thus keep the EKE estimates based on floe rotation variances only for those years and mooring locations for which there are over 50 floe observations (to have a reliable estimate of the variance of their rotation rates). Overall, there are 9 data points for which both the mooring data were available and a substantial amount of floes were available to compute their variance (Fig. 4). Most of the EKE estimates originate from mooring D which has the largest density of floe observations. Since both EKE estimates rely on calculating the variances (velocity variance for moorings and rotation rate variances for floes), we calculate their error bars as the 95% confidence intervals for estimating the variance of a normal distribution^48^.

While the majority of predicted and observed EKE points are close to the 1:1 line, there are clear outliers (Fig. 4, Supplementary Figures 6 and 7). All outliers are points with excessively strong observed EKE values marked as 233 (mooring D, year 2015), 95 (mooring D, year 2016), and 65 (mooring A, year 2007). Note, the numbers marking the points in Fig. 4 and Supplemenrary Fig. 6 simply denote the number of available floe observations in a given year and within a 200 km box centered at a given mooring location. The record of mooring D in 2015 is incomplete and contains only one eddy (the strongest one over all moorings). mooring D in 2016 starts with over half a year of missing data and ends with a few strong eddies (Supplementary Fig. 7d). Thus, the observed yearly mean EKE for these two points represents a highly-biased estimate of the spatial mean. The record of mooring A in year 2007 does not contain any significant gaps in observations or a rare case of an excessively strong eddy (Supplementary Fig. 7a); it being an outlier is possibly explained by an excessive number of eddies passing over the mooring location even though there was not necessarily an enhanced EKE in a large enough vicinity of the mooring. Finally, we note that the point marked 325 (mooring D, year 2012) is not an outlier but does contain some gaps in observations.

## Supplementary Information

Supplementary Information 1.

Supplementary Information 2.

## Data Availability

The library of satellite-derived sea ice floe observations and their rotation rates developed for this study is available at the Zenodo data repository^16^. All other datasets used in this study are publicly available and listed in Methods.
