# Supplementary material for: Spinning ice floes reveal intensification of mesoscale eddies in the western Arctic Ocean
Source: Sci Rep. 2022 Apr 29;12:7070. doi: 10.1038/s41598-022-10712-z (PMC9054753; doi:10.1038/s41598-022-10712-z)
Supplement: Supplementary file 1 — Supplementary Information 1. [file 41598_2022_10712_MOESM1_ESM.pdf]

Spinning ice floes reveal intensification of mesoscale eddies in the western Arctic Ocean: Supplementary Material

G. E. Manucharyan, R. Lopez-Acosta, M. M. Wilhelmus

1 Supplementary Video

Title: "Floe motion over an eddying ocean." The video shows the numerical simulation of a propagating sea ice floe subject to stresses from uniform atmospheric winds and an underlying oceanic mesoscale eddy field. Black arrows denote the surface ocean currents and colors represent their vorticity normalized by the Coriolis parameter. The angle relative to the initial orientation of the floe is shown in the center of the floe for each time frame. The simulation time in days is displayed as a title. Note how the instantaneous floe rotation is in the same direction as the rotation of eddies over which it passes, despite the presence of relatively strong winds of about 10 ms^{-1} that force the floe to translate from one eddy to another.

2 Floe observations in different years

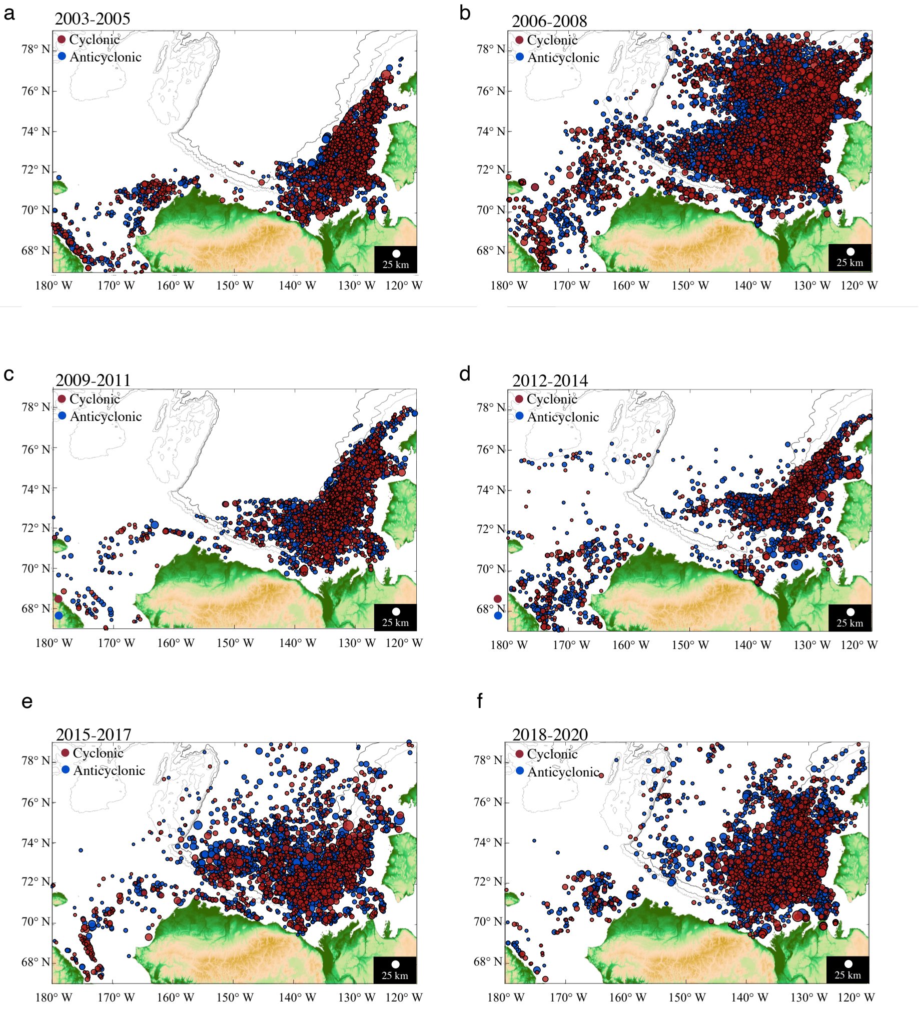

Figure 1. The normalized vorticity of sea ice floes employed in this study is plotted for the months of June and July for 2003-2011 (a-c), 2012-2017 (d-e), and 2018-20 (f). The red (blue) circles display cyclonic (anticyclonic) sea ice vorticity. Each marker specifies floe location with size being proportional to floe length-scale. The figure was plotted using MATLAB version R2021a www.mathworks.com

3 Motion of floes over an eddying ocean

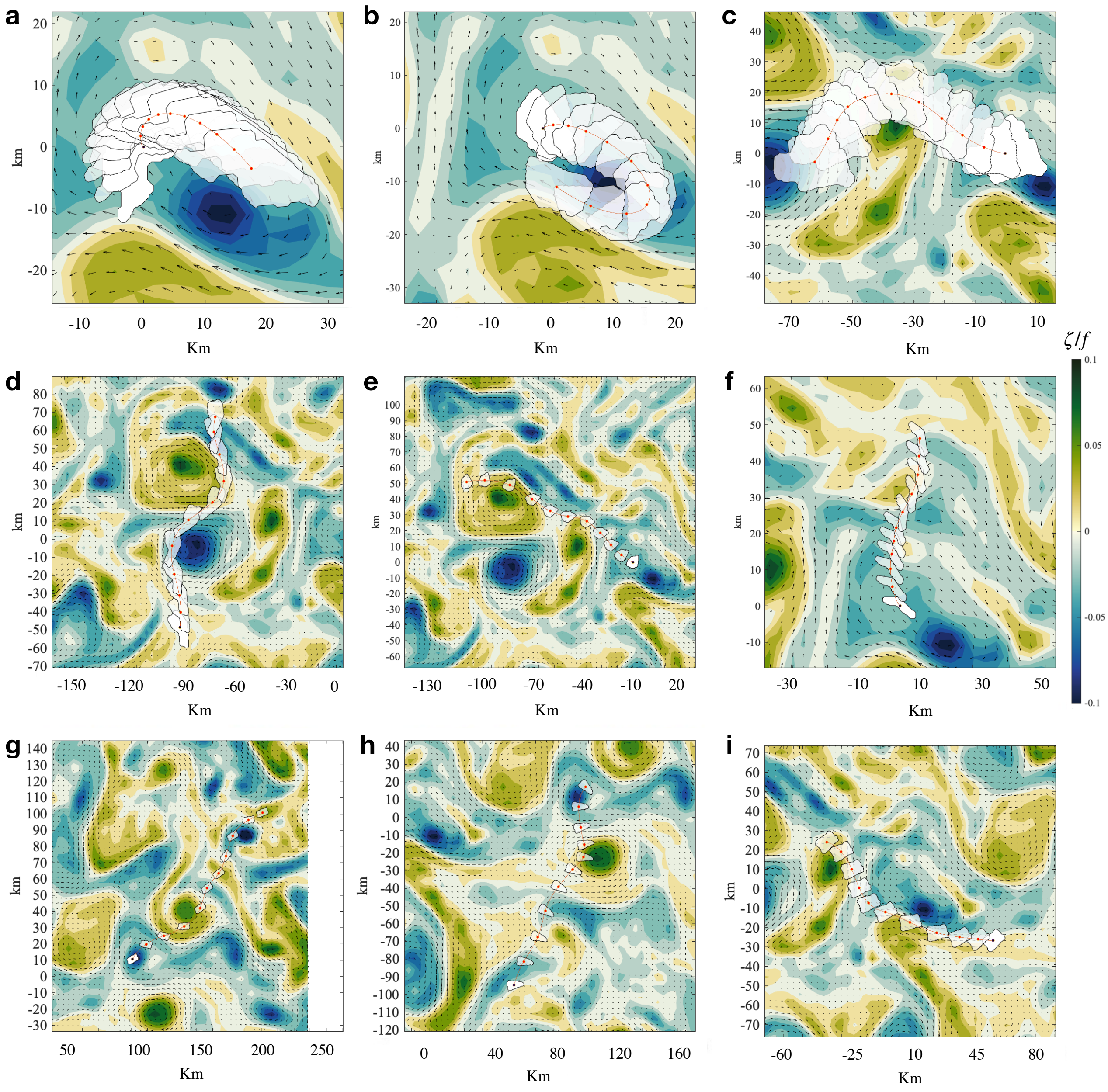

Figure 2. Motion of floes of different shapes and sizes subject to eddying ocean currents and uniform atmospheric winds. The color scheme corresponds to ocean vorticity values normalized by the Coriolis parameter. The direction of the associated ocean currents is represented with arrows. Ice fragments are plotted daily (in white). Lagrangian trajectories are plotted in red, with the initial position in black and subsequent locations of the centers of mass in red. The strength of the atmospheric wind fields varies (e.g., panels a,b, and c have relatively weak winds compared to the rest). The figure was plotted using MATLAB version R2021a `www.mathworks.com`

4 Quasigeostrophic equations

Following the notation of Arbic et al.[46], we write the two-layer quasigesotrophic equations for the perturbation variables around a uniform and steady background flow with zonal velocities (U_1, U_2) and associated mean potential vorticity gradients (Q_{1y}, Q_{2y}) in both layers. The perturbation potential vorticities (q_1, q_2) in the two layers are defined as:

$$q_1 = \nabla^2\psi_1 + \frac{\psi_2 - \psi_1}{(1+\delta)R_d^2} \quad \text{and} \quad q_2 = \nabla^2\psi_2 + \frac{\delta(\psi_1 - \psi_2)}{(1+\delta)R_d^2}, \tag{5}$$

where (ψ_1, ψ_2) are the perturbation streamfunctions in the top and bottom layer, $\delta = H_1/H_2$ is the ratio of layer depths, and R_d is the Rossby deformation radius. The mean potential vorticity gradients (Q_{1y}, Q_{2y}) are proportional to the mean velocity shear between the two layers:

$$Q_{1y} = \frac{U_1 - U_2}{(1+\delta)R_d^2} \quad \text{and} \quad Q_{2y} = \frac{\delta(U_2 - U_1)}{(1+\delta)R_d^2}. \tag{6}$$

Given the potential vorticity distributions in both layers, the corresponding streamfunctions can be uniquely computed – the inversion that in our numerical simulations is performed in spectral space. The conservation of potential vorticity presents the evolution equations:

$$q_{1t} + (u_1 + U_1)q_{1x} + v_1 q_{1y} = -v_1 Q_{1y} - \frac{C_d}{H_1}\nabla \times |\mathbf{u}_1|\mathbf{u}_1 + s.s.d. \tag{7}$$

$$q_{2t} + (u_2 + U_2)q_{2x} + v_2 q_{2y} = -v_2 Q_{2y} - r\nabla^2\psi_2 + s.s.d.\,, \tag{8}$$

where the left-hand-side denotes the inertial and non-linear advection terms, while the right-hand-side terms represent the effects of the eddy advection by the background potential vorticity gradients. The dissipation is due to the quadratic ice-ocean drag that is only present in the top layer and an Ekman-type friction in the bottom layer representing the weak energy drainage from the interactions with the abyssal layer that is not modeled here. The $s.s.d.$ label denotes the small-scale dissipation that is necessary to halt the forward-enstrophy cascade towards smaller scales; it is implemented in spectral space by filtering out high-wavenumber energy in the potential vorticity fields[46]. The domain size for numerical simulations is 400 km x 400 km, with double-periodic boundary conditions. The integration is performed in spectral space with 256 modes (corresponding to the spatial grid box size of 1.56 km). The simulations are performed until statistical equilibration in the eddy field is achieved (integration times range from a few years to a decade, depending on the model parameters).

The model parameters for the best-fit simulation are as follows: $R_d = 5.2$ km, $\delta = 1$, $U_1 - U_2 = 2.1$ cm s^{-1} (only the velocity shear matters for the development of instabilities), $C_d/H_1 = 2\times10^{-5}$ m^{-1} (assuming the effective drag coefficient $C_d = 2.5\times10^{-3}$), the linear dissipation timescale for the bottom layer $r = 0.01$ days^{-1}, $f = 10^{-4}$ s^{-1}, $\beta = 0$ (no beta effect). The mean flow is homogeneous throughout the whole double-periodic domain, and the resulting spatially-homogeneous eddy field is for evaluation of statistical rotational characteristics of the simulated floe motion.

5 Sensitivity of the eddy field and ice floe rotation to the QG model parameters

For each set of parameters $(\Delta U, R_d, \delta)$, we first run the QG model to obtain the statistically equilibrated samples of the eddy field (Fig. 3). Next, we use the eddy field to simulate the motion of sea ice floes (Fig. 2), which are also subject to atmospheric winds (taken from NCEP reanalysis and matching the time and the location of ice floe observations). Observed floes of various shapes and sizes are randomly initialized, with their trajectories and angular positions simulated for about half a day to a day to remove their dependence on the initial conditions (which lasts for a few hours). The rotation rates inferred by taking the 1-day finite-difference of the angular position of floes (mimicking what was done with satellite observations) closely match with an instantaneous rotation rate because it has persistence timescales of the order of a day to a few days. The instantaneous simulated rotation rates of ice floes are then used to compute the size dependence of the rotation rate variance. Finally, the best fit is found by adjusting the QG model parameters to minimize the error in the mismatch between the observed and simulated rotational variances (Fig. 4).

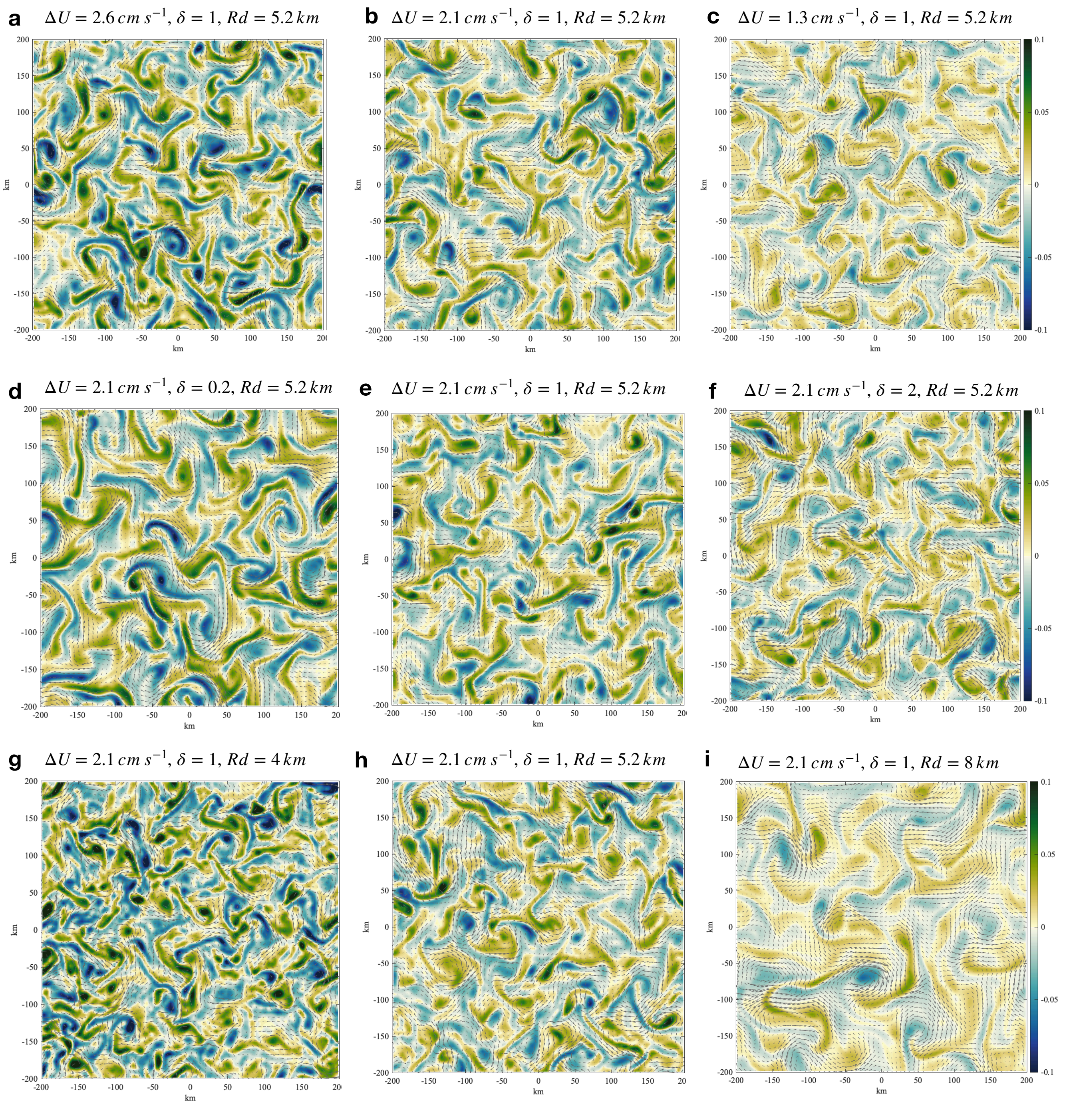

Figure 3. Examples of statistically equilibrated turbulent ocean eddy fields in the top layer for various parameters of the QG model. The color scheme corresponds to values of the ocean vorticity normalized by the Coriolis parameter. All panels have the same colorbar range. The direction of the associated ocean currents is represented with arrows. Parameter values are presented in panel titles with $\Delta U, Rd, \delta$ representing the bulk vertical shear of the horizontal velocity, Rossby deformation radius, and the ratio of layer depths, respectively. In each row, panels explore variations in a single parameter: panels a-c explore the parameter ΔU, panels d-f explore δ, and panels g-i explore Rd. Note that the three panels of the middle column correspond to different realizations of the eddy field with the same parameter set that is best-fitted to mimic the rotational statistics of the observed floes. The figure was plotted using MATLAB version R2021a www.mathworks.com

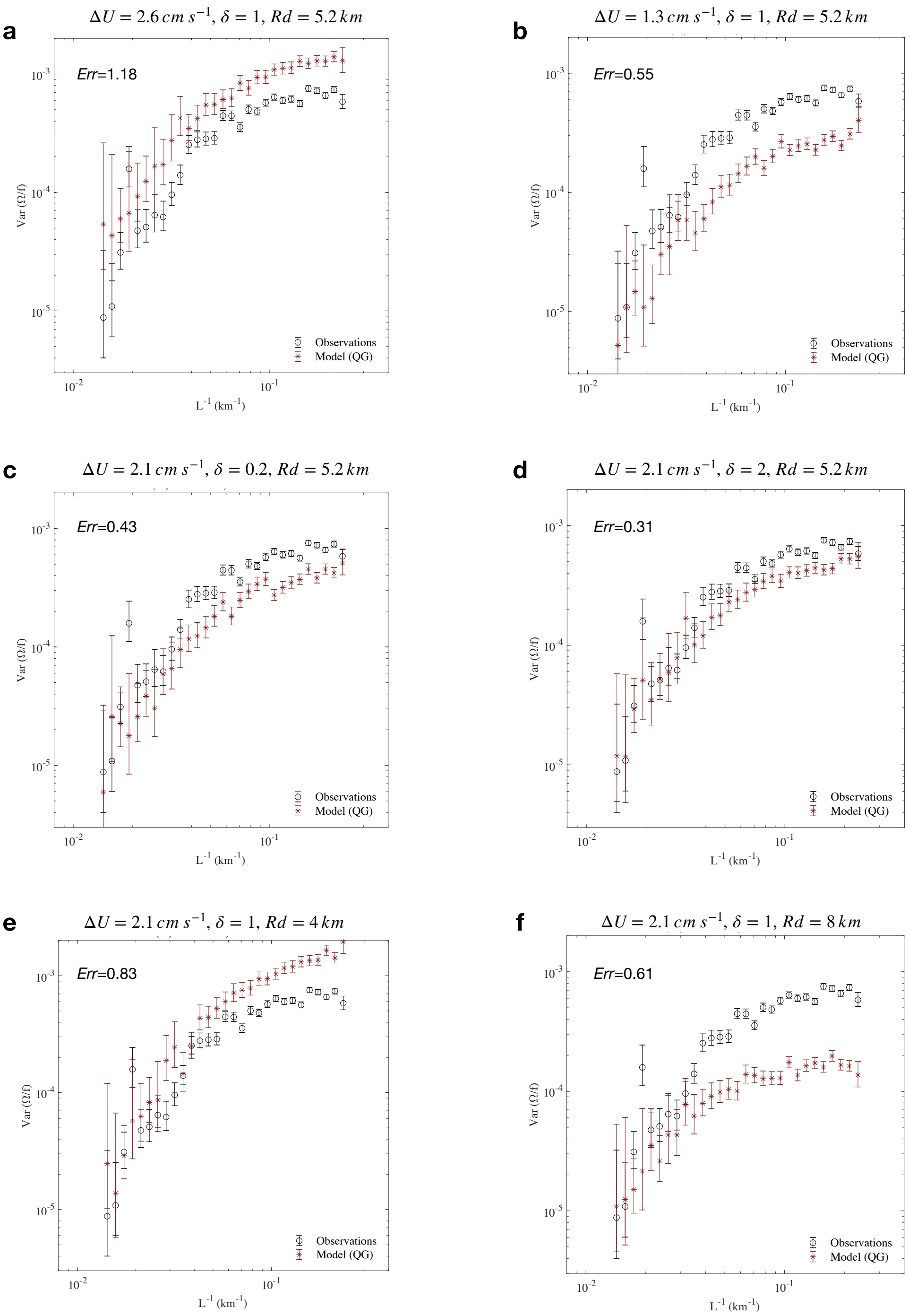

Figure 4. Vorticity variance plotted as a function of length-scale. Model results are shown in red. Observations are presented in black. Panels represent different parameter choices of the QG model (shown at titles), with the left and right columns corresponding to the eddy fields shown in the left and right columns of Supplementary Figure 3. The magnitude of the mismatch is quantified using the loss function Err (defined in Methods) and shown inside each panel. The variance figure for the best-fit parameters that minimize the loss function is shown in Fig. 3d ($Err = 0.19$).

6 Beaufort Gyre and its moorings

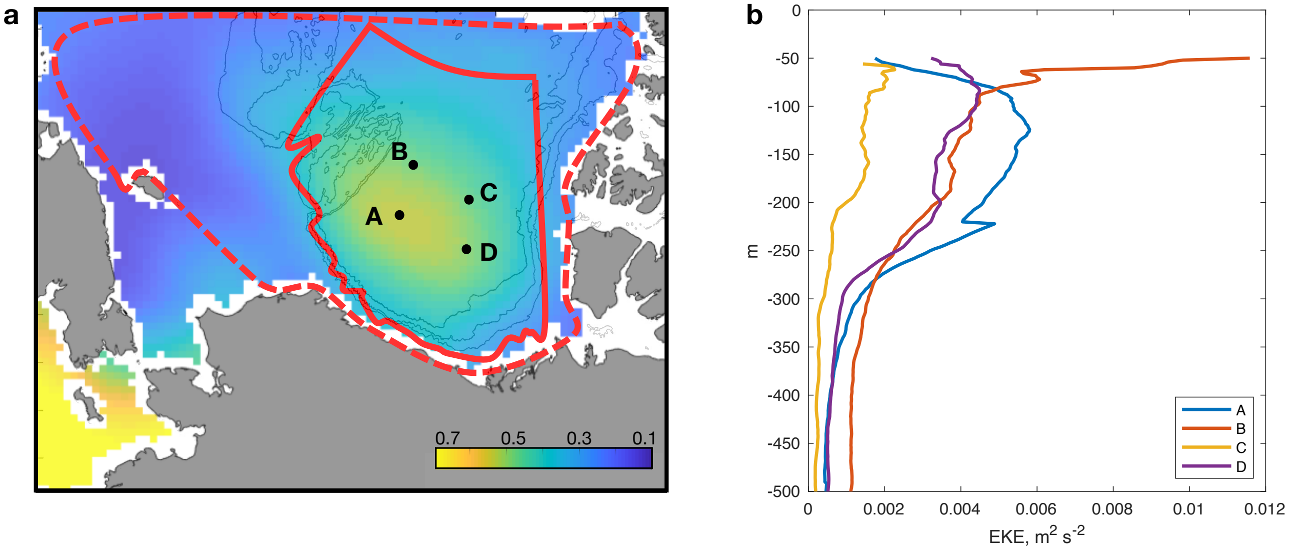

Figure 5. Region enclosing the Beaufort Gyre and the locations of the BGEP moorings. **a)** The region enclosing the Beaufort Gyre is marked by the thick red line. The location outside the Gyre used to compute the Sea Surface Height anomaly is delineated by the dotted red line. The SSH data is extracted from Armitage et al.[42]. The color indicates the May-July mean of the Dynamic Ocean Topography (2003 to 2014). The gray contour lines denote the 500 m, 1500 m, and 2500 m isobaths. The locations of the four BGEP moorings are marked with black dots. The figure was plotted using MATLAB version R2021a `www.mathworks.com`. **b)** The temporal mean eddy kinetic energy (EKE) calculated based on the entire available observational history of each BGEP mooring. For moorings A, C, and D, note the EKE enhancement in the subsurface layer ranging from about 100 to 200 m deep, and the EKE decrease towards the surface. Mooring B presents a qualitatively different EKE pattern with surface amplification, likely because it is adjacent to the sharp topographic feature, the Northwind ridge, and subject to topographic eddy generation. Most of the identified sea ice floes in our study correspond to the region near mooring D with an average subsurface EKE of about 4×10^{-3} m^2 s^{-2}.

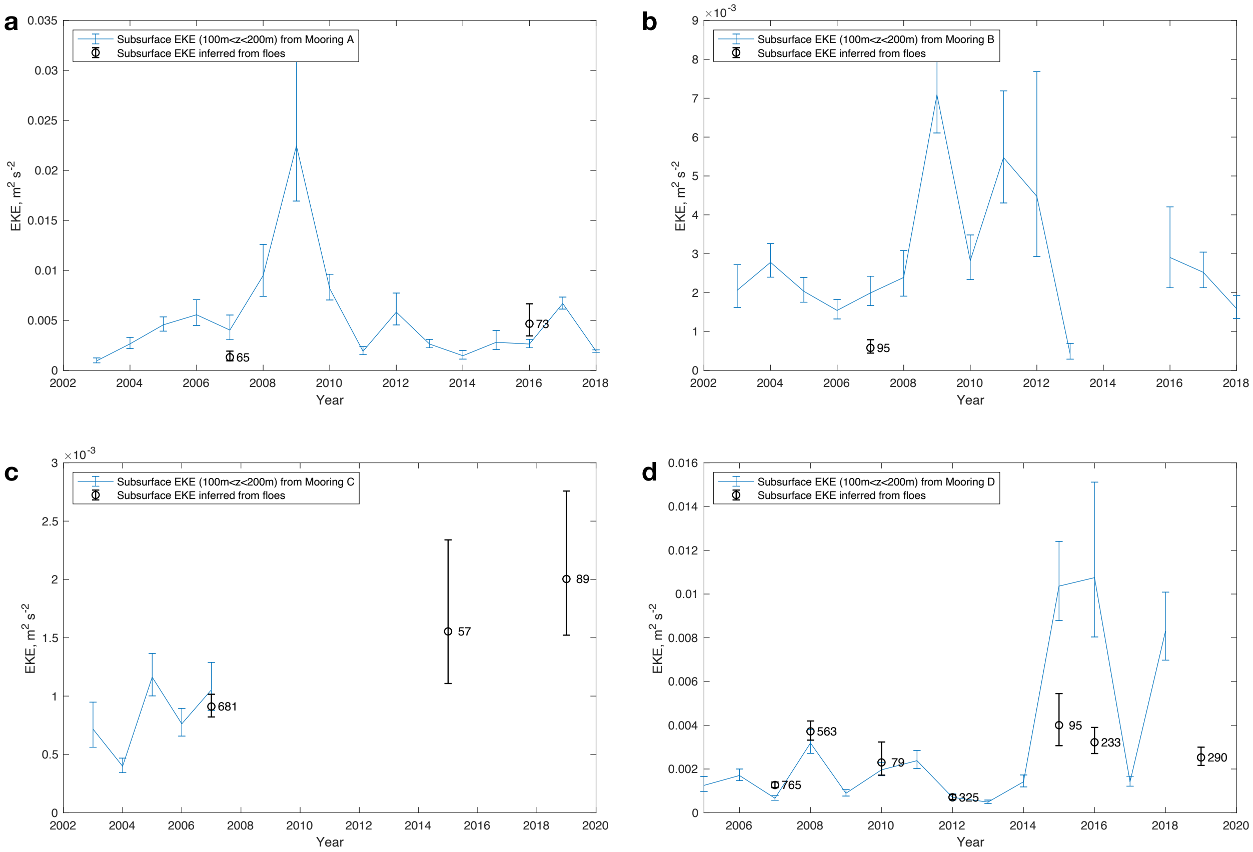

Figure 6. Comparison between the subsurface EKE from moorings (blue) and the EKE inferred from floe rotation in the vicinity of each mooring (black). Panels a-d correspond to the BGEP moorings A- D, respectively (locations shown in Supp. Fig. 5). The mooring EKEs are computed by first taking the horizontal velocity averages over a depth range of 100 m to 200 m and then computing their variances for each year of observations. The EKE estimated from the floes is calculated as $\alpha * Var(\Omega/f)$, where the parameter $\alpha = 8.5$ m^{2}s^{-2} is estimated based on the QG simulations. The numbers next to the EKE estimates represent the number of floes in a given year within a 200 km wide square box centered around the corresponding mooring location; EKE estimates are shown only for those years in which there were greater than 50 floes in the box. The EKE error bars represent the 95% confidence intervals in estimating the variances assuming a normal distribution for both eddy velocities and floe rotation rates.

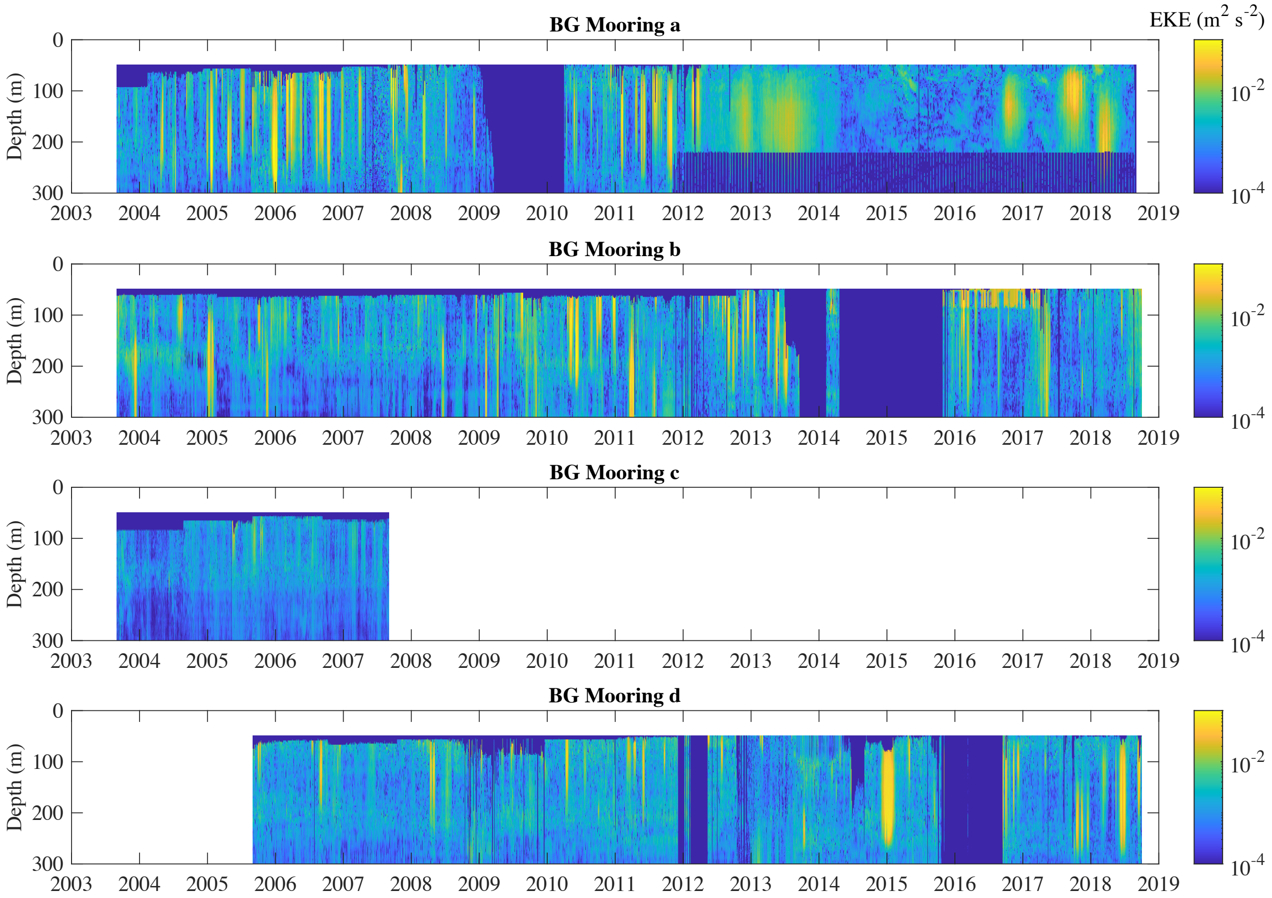

Figure 7. Time evolution of the eddy kinetic energy for the four BGEP moorings A,B,C, and D. The eddy kinetic energy is calculated with respect to the annual mean flow for each mooring. Saturated blue color represents missing or corrupt data.
